# Regional Differences in Prevalence of HIV-1 Discordance in Africa and Enrollment of HIV-1 Discordant Couples into an HIV-1 Prevention Trial

**DOI:** 10.1371/journal.pone.0001411

**Published:** 2008-01-09

**Authors:** Jairam R. Lingappa, Barrot Lambdin, Elizabeth Ann Bukusi, Kenneth Ngure, Linda Kavuma, Mubiana Inambao, William Kanweka, Susan Allen, James N. Kiarie, Joseph Makhema, Edwin Were, Rachel Manongi, David Coetzee, Guy de Bruyn, Sinead Delany-Moretlwe, Amalia Magaret, Nelly Mugo, Andrew Mujugira, Patrick Ndase, Connie Celum, for the Partners in Prevention HSV-2/HIV Transmission Study Group

**Affiliations:** 1 University of Washington, Department of Medicine, Seattle, Washington, United States of America; 2 University of Washington, Department of Epidemiology, Seattle, Washington, United States of America; 3 Center for Microbiology Research Kenya Medical Research Institute, Nairobi, Kenya; 4 Kenyatta National Hospital, Nairobi, Kenya; 5 Infectious Disease Institute, Makerere University Medical School, Kampala, Uganda; 6 Rwanda-Zambia HIV Research Group (RZHRG), Ndola, Zambia; 7 Rwanda-Zambia HIV Research Group (RZHRG), Kitwe, Zambia; 8 RZHRG and Emory University, Rollins School of Public Health, Atlanta, Georgia, United States of America; 9 University of Nairobi, Department of Obstetrics and Gynecology, Nairobi, Kenya; 10 Botswana-Harvard Partnership, Gaborone, Botswana; 11 Moi University, Department of Reproductive Health, Eldoret, Kenya; 12 Kilimanjaro Christian Medical Centre, Moshi, Tanzania; 13 Infectious Disease Epidemiology Unit, University of Cape Town, Cape Town, Republic of South Africa; 14 Perinatal HIV Research Unit, University of Witwatersrand, Johannesburg, Republic of South Africa; 15 Reproductive Health Research Unit, University of Witwatersrand, Johannesburg, Republic of South Africa; 16 University of Washington, Department of Laboratory Medicine, Seattle, Washington, United States of America; Institute of Human Virology, United States of America

## Abstract

**Background:**

Most HIV-1 transmission in Africa occurs among HIV-1-discordant couples (one partner HIV-1 infected and one uninfected) who are unaware of their discordant HIV-1 serostatus. Given the high HIV-1 incidence among HIV-1 discordant couples and to assess efficacy of interventions for reducing HIV-1 transmission, HIV-1 discordant couples represent a critical target population for HIV-1 prevention interventions and prevention trials. Substantial regional differences exist in HIV-1 prevalence in Africa, but regional differences in HIV-1 discordance among African couples, has not previously been reported.

**Methodology/Principal Findings:**

The Partners in Prevention HSV-2/HIV-1 Transmission Trial (“Partners HSV-2 Study”), the first large HIV-1 prevention trial in Africa involving HIV-1 discordant couples, completed enrollment in May 2007. Partners HSV-2 Study recruitment data from 12 sites from East and Southern Africa were used to assess HIV-1 discordance among couples accessing couples HIV-1 counseling and testing, and to correlate with enrollment of HIV-1 discordant couples. HIV-1 discordance at Partners HSV-2 Study sites ranged from 8–31% of couples tested from the community. Across all study sites and, among all couples with one HIV-1 infected partner, almost half (49%) of couples were HIV-1 discordant. Site-specific monthly enrollment of HIV-1 discordant couples into the clinical trial was not directly associated with prevalence of HIV-1 discordance, but was modestly correlated with national HIV-1 counseling and testing rates and access to palliative care/basic health care (r = 0.74, p = 0.09).

**Conclusions/Significance:**

HIV-1 discordant couples are a critical target for HIV-1 prevention in Africa. In addition to community prevalence of HIV-1 discordance, national infrastructure for HIV-1 testing and healthcare delivery and effective community outreach strategies impact recruitment of HIV-1 discordant couples into HIV-1 prevention trials.

## Introduction

Among the estimated 40 million people worldwide currently living with HIV-1 infection, 65% reside in sub-Saharan Africa [1], with heterosexual exposure as the primary mode of HIV transmission. Prior studies have found high rates of HIV-1 transmission occurring between HIV-discordant partners (where one partner is HIV- infected and one is HIV-uninfected) who are often in stable partnerships but unaware of both partner's HIV-1 serostatus [2]–[4]. Recent data from Uganda indicate that 83% of HIV-infected men and 77% of HIV-infected women are or have been married [5]. Furthermore, among HIV-1 infected married persons, 75% of men and 96% of women report having had sex only with their spouse [5]–[7]. HIV-1 counseling and testing specifically adapted to couples including education about HIV-1 discordance rather than an individual-focused HIV-1 risk assessment and counseling can help reduce HIV-1 transmission [2], [8], [9]. This underscores that stable HIV-1-discordant African couples are a critical target for counseling and testing and evaluation of new prevention interventions.

Microbicides, vaccines, pre-exposure prophylaxis, male circumcision, HSV-2 suppression, and other interventions are currently under evaluation to increase the range of public health tools for HIV-1 prevention. Efficacy trials and public health implementation studies of these interventions will require tens of thousands of participants over the coming years. Prevention trials that recruit and follow HIV-1 discordant couples are of particular importance in evaluating interventions for reducing HIV-1 infectiousness while also permitting evaluation of biologic and behavioral factors in both the transmitting and susceptible host which modulate the risk of HIV-1 transmission.

There are unique challenges to conducting clinical trials with HIV-1-discordant couple cohorts. In particular, recruiting both partners to undergo HIV-1 counseling and testing together is difficult, as only 10–30% of persons in Africa who are tested for HIV-1 come as a couple [10]. This is in part due to the reluctance of men to be tested for HIV-1 in many parts of Africa, coupled with assumptions that their partner's HIV-1 status is the same as theirs (which is referred to as ‘HIV-1 testing by proxy’). In addition, VCT counselors typically trained in individual VCT techniques are reluctant to engage in couples' HIV-1 counseling and testing (CHCT) due to additional time required, for CHCT, skills needed for addressing other relationship issues that may be raised, and concerns about as well as the risk of domestic violence, abandonment or stigmatization if one but not both partners learn that they are HIV-1-infected [11]–[14]. Importantly, many HIV-1 counselors and couples have myths, misperceptions, and denial about HIV-1 discordance [9].

Between November 2004 and May 2007 we recruited a cohort of 3408 HIV-1-discordant couples from 14 sites in eastern and southern Africa into the Partners in Prevention HSV-2/HIV-1 Transmission Trial (“Partners HSV-2 Study”). Recruitment for this trial provided a unique opportunity to evaluate the prevalence of HIV-1 discordance in diverse African communities and identify factors correlating with recruitment of HIV-1-discordant couples.

## Methods

The Partners HSV-2 Study is designed to assess the impact of HSV-2 suppression with acyclovir (400 milligrams orally, twice daily) compared to placebo in reducing HIV-1-transmission among couples in which one partner is HIV-1/HSV-2 co-infected with a CD4 count ≥250 and not eligible for antiretroviral initiation, and their partner is HIV-1-uninfected (ClinicalTrials.gov ID # NCT00194519) [15]–[17]. In May 2007, 14 clinical trial sites in 7 countries in Eastern and Southern Africa completed screening of 7863 HIV-1 discordant couples and enrollment of 3408 HIV-1-discordant couples. Follow-up will continue through May 2008 to evaluate the efficacy of herpes suppression on reducing HIV-1 transmission to the initially HIV-1 uninfected partner.

### Partners HSV-2 Study sites

Twelve of 14 Partners HSV-2 Study clinical trial sites recruited couples from the community; two sites (Kigali, Rwanda and Lusaka, Zambia) recruited couples from existing cohorts of HIV-1 discordant couples and therefore are not included in this analysis. Of the 12 sites, six are in Eastern Africa, including Nairobi, Thika, Kisumu and Eldoret, Kenya; Kampala, Uganda; and Moshi/Arusha, Tanzania; and six in Southern Africa, including Soweto, Orange Farm and Gugulethu, South Africa; Ndola and Kitwe, Zambia; and Gaborone, Botswana.

### Partners HSV-2 Study recruitment methods

#### Couples HIV-1 Counseling and Testing (CHCT)

All study sites used CHCT as the initial entry point for identifying discordant couples who could then be evaluated for study eligibility. Over 1000 community-based and study site counselors were trained through the Partners HSV-2 Study using a CDC, NIMH, Liverpool School of Tropical Medicine standardized CHCT curriculum that covered basic counseling methods, the importance of HIV-1-discordance, review of HIV-1 risk issues for couples and approaches to counseling couples testing concordant HIV-1-positive, concordant HIV-1-negative or HIV-1-discordant.

#### Partners HSV-2 Study Community Recruitment

Couples were either recruited directly through community outreach from the Partners HSV-2 Study clinic including methods similar to those reported elsewhere [2], [8], [18], [19], or indirectly through training of hundreds of community counselors in CHCT skills and collaboration with community voluntary counseling and testing (VCT) centers, antenatal and HIV-1 clinics, non-governmental organizations, and coupled with direct community outreach efforts with street theatre, one-to-one peer outreach, radio discussions, and other messages about CHCT in the community. These multiple strategies led to referral of interested HIV-1 discordant couples to the Partners HSV-2 Study clinics for additional counseling about HIV-1 discordance and screening for eligibility for the Partners HSV-2 Study.

### CHCT and VCT Data Collection

Community-based CHCT HIV-1 testing data among couples was obtained from the principal community VCT centers who referred HIV-1-discordant couples to the Partners HSV-2 Study clinics and from testing of couples of unknown HIV-1 status at the Partners HSV-2 Study clinics. These data were collected at the Ndola and Kitwe, Zambia study sites from July 2005 through April 2006. For the remaining sites due to the additional coordination and efforts involved in data collection from multiple referring VCT centers, prescreening data was only collected between December 2006 and April 2007.

Prescreening data were used to calculate HIV-1 discordance, HIV-1 negative and HIV-1 positive concordance as a percentage of the total number of couples tested at those sites during the data collection time period and as a weighted regional HIV-1 discordance rate across sites on a country-specific basis, regionally (Eastern and Southern Africa) and across these 12 Partners HSV-2 Study sites.

As an indicator of the utilization of HIV-1 counseling and testing and basic health care services in participating countries, we used publicly accessible data from the United States President's Emergency Program For AIDS Relief (PEPFAR) [20]. We defined the National HIV-1 Response Index (NHRI) as the sum of the annual number of individuals receiving HIV-1 counseling and testing and palliative and basic health care services (including TB/HIV-1) accessed in a country normalized to the estimated number of people living with HIV-1/AIDS (PLWHA).

## Results

Descriptive data for the 12 Partners HSV-2 Study sites participating in community-based recruitment of HIV-1-discordant couples are presented in Table 1. Study sites vary from high population-density cities and associated townships to smaller rural towns. Based on Partners HSV-2 Study prescreening data, the prevalence of stable HIV-1-discordant partnerships ranged from 8 to 31% of total couples tested at each study site. Overall, Eastern and Southern African sites reported 12% and 18% HIV-1 discordance, respectively, with 15% HIV-1 discordance found across the whole study. Among all couples tested who had at least one HIV-1 infected partner, the proportion of couples which were HIV-1-discordant varied by study sites from 36-85% with an overall rate of 49%.

**Table 1:** PreScreening Data and Other Characteristics of Partners HSV-2 Study Sites

Country	Site	Local Pop*	HIV-1 Prevalence (%)		Couples Tested&	HIV-1 Testing Results			HIV-1 Discordance Prevalence (% Couples Tested)	National HIV-1 Response Index (NHRI)@	Partners HSV-2 Study Monthly Enrollment
			Site#	Country**		Discordant	Concordant				
							Positive	Negative			
**Kenya**	Nairobi	4,000,000	9.9	6.1	624	84 (13)	49 (8)	491 (79)	13.6	0.81	15.9
	Kisumu ˆ	320,000	15		3237	500 (15)	698 (22)	2039 (63)			
	Thika	448,000	6–9		751	64 (9)	23 (3)	664 (88)			
	Eldoret ˆ	450,000	6–9		383	29 (8)	40 (10)	314 (82)			
**Uganda**	Kampala	1,200,000	13	6.7	2079	181 (9)	183 (9)	1715 (82)	8.7	1.29	17.3
**Tanzania**	Moshi/Arusha	145,000	9.6	6.5	477	56 (12)	10 (2)	411 (86)	11.7	0.57	9.3
**S. Africa**	Soweto	1,200,000	30	18.8	286	66 (23)	62 (22)	158 (55)	27.4	0.36	8.2
	Orange Farm	500,000	30		525	145 (28)	89 (17)	290 (55)			
	Gugulethu	340,000	27		315	97 (31)	55 (17)	163 (52)			
**Zambia**	Ndola	636,000	20	17	2241	271 (12)	474 (21)	1496 (67)	14.2	0.29	12.5
	Kitwe	675,000	20		2827	450 (16)	628 (22)	1749 (62)			
**Botswana**	Gaborone	208,000	24	24.1	2589	522 (20)	241(9)	1826 (71)	20.1	1.04	12.8

* 2004 or 2005 census data.

** From PEPFAR (http//www.pepfar.gov/): “National HIV-1 prevalence among adults aged 15–49” for each country listed.

ˆ Clinical trial recruitment also extended to outlying districts with total population of 0.75–1.5 million persons.

# Source of HIV-1 prevalence data: Kenya-reference [23]; Uganda–reference [7]; Tanzania–reference [24]; S. Africa–reference [25]; Zambia–reference [26]; Botswana–reference [27].

& -Total number of couples receiving HIV counseling and testing during previously defined recruitment periods: July 2005–April 2006 (Ndola and Kitwe, Zambia) and December 2006–April 2007 (all other Partners HSV-2 Study sites).

@ -NHRI calculated from PEPFAR data (http//www.pepfar.gov/) as: (“# individuals receiving counseling and testing in settings other than PMTCT in FY2006”+“# HIV-1 infected individuals receiving palliative care/basic health care and support in FY2006 (including HIV-1/TB)”)/“Adults and children (age 0–49) living with HIV-1 at the end of 2005”.

Approximately, 51,900 couples were prescreened for HIV-1 serostatus by partnering collaborating VCT centers, clinics or at the Partners HSV-2 Study clinics. From this large community CHCT effort, 7863 HIV-1-discordant couples were referred to Partners HSV-2 Study sites and screened for study eligibility, of whom 4178 met study eligibility criteria; 3408 couples were enrolled into the Partners HSV-2 Study. Thus, for each HIV-1-discordant couple enrolled in the clinical trial, over 15 couples (site range 4.4–42.1) were provided CHCT and learned their HIV-1 status and 1.9 HIV-1-discordant couples (site range 1.5–2.3) were screened for study eligibility.

Despite HIV-1 discordance being more prevalent in Southern Africa, the site-specific Partners HSV-2 Study monthly enrollment rate was higher in Eastern compared to Southern African sites (15.1 versus 11.2 couples/month, respectively). Overall, HIV-1 discordance rates among couples tested in the community were not significantly correlated with site-specific monthly study enrollment rates (correlation coefficient -0.64, p = 0.16). There was a trend for an association between the country NHRI (Table 1) and average monthly site-specific enrollment of HIV-1 discordant couples into the Partners HSV-2 Study (correlation coefficient = 0.74, p = 0.09). Other PEPFAR-reported measures for dissemination of HIV-1 prevention information, such as persons accessing ABC (‘Abstinence, 'Be faithful and use Condoms’) messages, as measured by PEPFAR, did not strongly correlate with study enrollment (correlation coefficient = 0.32, p = 0.54, data not shown).

## Discussion

HIV-1 discordance was highly prevalent in the 12 communities from Eastern and Southern Africa that participated in this multi-site clinical trial of HSV-2 suppression to prevent HIV-1 transmission within HIV-1 discordant couples. Overall, among couples presenting for HIV-1 testing in which one partner was infected with HIV-1, 49% were identified as HIV-1-discordant. The prevalence of HIV-1 discordance has been documented previously in several African countries including in Zambia [21], Uganda [3], Kenya [22]. Significant regional differences in HIV-1 discordance have previously been reported among couples identified in the cross-sectional Four Cities study [22]. Notably, two sites, Ndola, Zambia and Kisumu, Kenya were included in both the Partners HSV-2 Study and the Four Cities Study and observed to have similar prevalence of HIV-1 discordance (12% versus 13% in Ndola and 16% versus 23% in Kisumu) 10 years apart and using different sampling methodologies. The continued high prevalence of HIV-1 discordance in diverse African sites underscores the public health importance of couples with HIV-1 discordant results for HIV-1 prevention. By facilitating CHCT training for almost 1000 counselors and heightening community awareness of HIV-1-discordance in 14 study sites located in seven countries, the Partners HSV-2 Study has demonstrated the feasibility of recruiting and counseling HIV-1 discordant couples in diverse regions of Africa. Building capacity for CHCT, with development of messages about testing as couples and the importance of HIV-1 discordance in HIV-1 transmission, is an important and sustainable public health initiative in Africa.

Interestingly, we found no statistically significant association between Partners HSV-2 Study enrollment rates and prevalence of HIV-1 discordance among couples in study site communities. We did find a trend for an association between site-specific average monthly study enrollment and the NHRI, a normalized measure of testing and care services accessed in a country. While difficulties in mobilizing men to be tested, HIV-1-associated stigma, and fears associated with disclosure of HIV-1 status remain ongoing challenges in recruiting HIV-discordant couples, the Partners HSV-2 Study recruitment experience suggests that enrolling HIV-1 discordant couples for a clinical trial is affected more by the availability of HIV-1 counseling, testing and palliative and basic healthcare services and support for HIV-infected persons than by a high prevalence of HIV-1 discordance. Notably, levels of community access to “ABCs” messages, as measured by PEPFAR, did not have an association with enrollment of HIV-1-discordant couples. This may arise from abstinence and condom use messages being viewed as less relevant to stable couples.

HIV-1 prevention trials involving HIV-1 discordant couples can be implemented in countries with less developed VCT infrastructure and lower HIV-1 testing rates. The community recruitment strategy utilized in the Ndola and Kitwe, Zambia sites, based on a model developed in Rwanda [2] and previously implemented in Lusaka, Zambia[21], was associated with high rates of enrollment despite relatively lower national levels of counseling and testing in Zambia. However, local factors impact the effectiveness of specific clinical trial recruitment strategies, and attempts to implement this recruitment model in other Partners HSV-2 Study sites were less successful than in Zambia. Furthermore, recruitment costs were reduced if referrals were obtained from community VCTs.

In conclusion, HIV-1 discordant couples represent an important target population for HIV-1 prevention, given the high rates of HIV-1 transmission in couples that do not know or disclose their HIV-1 status. HIV-1 discordant couples are also a valuable population for clinical trials evaluating vaccines, microbicides and other HIV-1 prevention interventions. Our findings suggest that increased national access to HIV-1 counseling and testing services and health care can facilitate identification of HIV-1 discordant couples for prevention interventions and enrollment into HIV-1 prevention trials. In view of these public health benefits and the importance of identifying new prevention strategies to reduce HIV-1 transmission in Africa, it is of critical importance to enhance community mobilization to encourage couples to be tested for HIV-1 as couples rather than as individuals, expand training of counselors in CHCT techniques and counseling messages about HIV-1 discordance, and prioritize prevention for HIV-1-discordant couples in Africa.

## References

[1] UN AIDS/World Health Organization (2006). AIDS Epidemic Update..

[2] Allen S, Tice J, Van de Perre P, Serufilira A, Hudes E (1992). Effect of serotesting with counselling on condom use and seroconversion among HIV discordant couples in Africa.. Bmj.

[3] Carpenter LM, Kamali A, Ruberantwari A, Malamba SS, Whitworth JA (1999). Rates of HIV-1 transmission within marriage in rural Uganda in relation to the HIV sero-status of the partners.. Aids.

[4] Hira SK, Nkowane BM, Kamanga J, Wadhawan D, Kavindele D (1990). Epidemiology of human immunodeficiency virus in families in Lusaka, Zambia.. J Acquir Immune Defic Syndr.

[5] Baryarama F, Bunnell R, McFarland W, Hudes ES, Neilands TB (2007). Estimating HIV incidence in voluntary counseling and testing clients in Uganda (1992–2003).. J Acquir Immune Defic Syndr.

[6] Bunnell RE, Opio A, Musinguzi J, Kirungi W, Ekwaru P (2008). HIV transmission risk behavior among HIV-infected adults in Uganda: results of a nationally representative survey.. AIDS In press.

[7] Ministry of Health [Uganda] and ORC Macro (2006). Uganda HIV/AIDS Sero-Behavioural Survey 2004-5. Calverton, Maryland, USA..

[8] Allen S, Meinzen-Derr J, Kautzman M, Zulu I, Trask S (2003). Sexual behavior of HIV discordant couples after HIV counseling and testing.. Aids.

[9] Bunnell RE, Nassozi J, Marum E, Mubangizi J, Malamba S (2005). Living with discordance: knowledge, challenges, and prevention strategies of HIV-discordant couples in Uganda.. AIDS Care.

[10] Baryarama F, Bunnell RE, Ransom RL, Ekwaru JP, Kalule J (2004). Using HIV voluntary counseling and testing data for monitoring the Uganda HIV epidemic, 1992-2000.. J Acquir Immune Defic Syndr.

[11] Antelman G, Smith Fawzi MC, Kaaya S, Mbwambo J, Msamanga GI (2001). Predictors of HIV-1 serostatus disclosure: a prospective study among HIV-infected pregnant women in Dar es Salaam, Tanzania.. Aids.

[12] Gaillard P, Melis R, Mwanyumba F, Claeys P, Muigai E (2002). Vulnerability of women in an African setting: lessons for mother-to-child HIV transmission prevention programmes.. Aids.

[13] Nebie Y, Meda N, Leroy V, Mandelbrot L, Yaro S (2001). Sexual and reproductive life of women informed of their HIV seropositivity: a prospective cohort study in Burkina Faso.. J Acquir Immune Defic Syndr.

[14] Porter L, Hao L, Bishai D, Serwadda D, Wawer MJ (2004). HIV status and union dissolution in sub-Saharan Africa: the case of Rakai, Uganda.. Demography.

[15] Celum C, Levine R, Weaver M, Wald A (2004). Genital herpes and human immunodeficiency virus: double trouble.. Bull World Health Organ.

[16] Corey L, Wald A, Celum CL, Quinn TC (2004). The effects of herpes simplex virus-2 on HIV-1 acquisition and transmission: a review of two overlapping epidemics.. J Acquir Immune Defic Syndr.

[17] Wald A (2004). Synergistic interactions between herpes simplex virus type-2 and human immunodeficiency virus epidemics.. Herpes.

[18] Allen SEC, Karita E ea (2007). Promotion of Couples Voluntary Counselling and Testing for HIV Through Influential Networks in Two African Capital Cities.. Biomed Central In Press.

[19] Chomba E, Allen S, Kanweka W, Tichacek A, Cox G (2007). Evolution of Couples' Voluntary Counseling and Testing for HIV in Lusaka, Zambia.. J Acquir Immune Defic Syndr In Press.

[20] Office of United States Global AIDS Coordinator (2007). The United States President's Emergency Plan for AIDS Relief: 2007 Country Profiles..

[21] McKenna SL, Muyinda GK, Roth D, Mwali M, Ng'andu N (1997). Rapid HIV testing and counseling for voluntary testing centers in Africa.. Aids 11 Suppl.

[22] Freeman EE, Glynn JR, Study Group on Heterogeneity of HIV Epidemics in African Cities (2004). Factors affecting HIV concordancy in married couples in four African cities.. AIDS.

[23] Kenyan Ministry of Health and Central Bureau of Statistics (2003). Kenya Demographic and Health Survey. Nairobi, Kenya..

[24] Kapiga SH, Sam NE, Mlay J, Aboud S, Ballard RC (2006). The epidemiology of HIV-1 infection in northern Tanzania: results from a community-based study.. AIDS Care.

[25] Dorrington R (2006). The Demographic Impact of HIV/AIDS in S. Africa Center for Actuarial Research, S. African MRC and Actuarial Society of S. Africa..

[26] Zambia Central Statistics Office (2003). Zambia Demographic and Health Survey 2001–2002. Zambia Central Statistics Office..

[27] UN AIDS/World Health Organization (2006). Uniting the World Against AIDS-Botswana. UNAIDS/WHO..

